# Biocontrol Traits Correlate With Resistance to Predation by Protists in Soil Pseudomonads

**DOI:** 10.3389/fmicb.2020.614194

**Published:** 2020-12-15

**Authors:** Nathalie Amacker, Zhilei Gao, Betina C. Agaras, Ellen Latz, George A. Kowalchuk, Claudio F. Valverde, Alexandre Jousset, Simone Weidner

**Affiliations:** ^1^Ecology and Biodiversity Group, Institute of Environmental Biology, University of Utrecht, Utrecht, Netherlands; ^2^Laboratorio de Fisiología y Genética de Bacterias Beneficiosas para Plantas, Departamento de Ciencia y Tecnología, Centro de Bioquímica y Microbiología del Suelo, Universidad Nacional de Quilmes, Buenos Aires, Argentina; ^3^German Centre for Integrative Biodiversity Research (iDiv), Halle-Jena-Leipzig, Leipzig, Germany; ^4^Department of Microbial Ecology, Netherlands Institute of Ecology, Wageningen, Netherlands

**Keywords:** rhizobacteria, PGPR, protozoa, multitrophic interactions, biocontrol

## Abstract

Root-colonizing bacteria can support plant growth and help fend off pathogens. It is clear that such bacteria benefit from plant-derived carbon, but it remains ambiguous why they invest in plant-beneficial traits. We suggest that selection via protist predation contributes to recruitment of plant-beneficial traits in rhizosphere bacteria. To this end, we examined the extent to which bacterial traits associated with pathogen inhibition coincide with resistance to protist predation. We investigated the resistance to predation of a collection of *Pseudomonas* spp. against a range of representative soil protists covering three eukaryotic supergroups. We then examined whether patterns of resistance to predation could be explained by functional traits related to plant growth promotion, disease suppression and root colonization success. We observed a strong correlation between resistance to predation and phytopathogen inhibition. In addition, our analysis highlighted an important contribution of lytic enzymes and motility traits to resist predation by protists. We conclude that the widespread occurrence of plant-protective traits in the rhizosphere microbiome may be driven by the evolutionary pressure for resistance against predation by protists. Protists may therefore act as microbiome regulators promoting native bacteria involved in plant protection against diseases.

## Introduction

Plant-associated microorganisms are an essential component of plant growth and health (Berendsen et al., 2012; Mendes et al., 2013). Plant roots are in particular a hot-spot of plant-microbe interactions, with root-associated microorganisms modulating plant hormonal balance (Lugtenberg and Kamilova, 2009; Ravanbakhsh et al., 2018) and plant immune responses (van Loon, 2007; Zamioudis and Pieterse, 2012). Microbe-microbe interactions are also linked to plant health, as plant-associated bacteria are known to be able to protect plants against pathogens by producing inhibitory secondary metabolites or competing for resources (Raaijmakers et al., 2009; Gu et al., 2020). The participation of microorganisms to plant health can contribute to a natural immunity of soils (Cook et al., 1995; Weller et al., 2002) and thereby reduce the need for environmentally harmful pesticides (e.g., Fravel, 2005; Haas and Défago, 2005). Although many root-associated microorganisms have the potential to protect plants, these activities can be highly variable, and we still have little information regarding drivers affecting pathogen-suppressive microbes. In natural systems, plant-associated microorganisms face multiple biotic interactions that constrain their fitness (Finkel et al., 2017; Wallenstein, 2017; Sessitsch et al., 2019). One particularly strong fitness pressure is that imposed by predation by free-living, phagotrophic protists. These act as key regulators of rhizosphere microbiome assembly through their intense and selective predatory activity (Gao et al., 2019).

Protists are a paraphyletic group encompassing most micro-eukaryotes and are present in the soil at densities in the range of 10^4^ individuals per gram (Adl et al., 2018; Geisen et al., 2018). Covering a wide range of sizes, typically from micrometers to few millimeters (Geisen et al., 2017), and morphotypes (e.g., testate and naked amoeba, flagellates, ciliates), protists occupy numerous ecological niches within the soil food web (Geisen et al., 2018). In line with their high taxonomic and functional diversity, protist species differ widely in their feeding behavior (Weekers and Van Der Drift, 1993; Jürgens and Matz, 2002). For instance, different morphotypes vary in their ability to physically reach their prey. While flagellates mainly feed by filtering the liquid around them using their flagellum (Boenigk et al., 2001), amoeba glide on surfaces, using their pseudopods to reach small cavities (Anderson, 2016). Prey selection goes even beyond discrimination based solely on physical accessibility: some protists can further select their bacterial prey based on their size and cell surface biochemistry (John and Davidson, 2001; Wootton et al., 2007). Thus, predation by protists acts as a selective pressure on bacterial communities, and this predatory pressure depends on the protist species. Interestingly, while closely related protists can in some cases elicit similar changes in the composition of the bacterial communities, in other cases they induce highly disparate modifications (Glücksman et al., 2010; Pedersen et al., 2011).

In order to escape predation, bacteria have developed a range of defense mechanisms. Common strategies include morphological changes such as filament formation, size shifts (Güde, 1979), but also behavioral and physiological changes such as biofilm formation (Raghupathi et al., 2018), enhanced motility (Matz and Jürgens, 2003) and/or the production of inhibitory compounds (Matz and Kjelleberg, 2005). While these adaptations have been mostly studied and reported in aquatic systems (Hahn and Höfle, 2001; Pernthaler, 2005), they may be of direct relevance for soil and rhizosphere microbiome functioning. From a plant perspective, indeed, some traits conferring resistance to protists also contribute to disease suppression. Indeed, numerous compounds produced by soil pseudomonads that have long been known for their antifungal activity, such as 2,4-diacetylphloroglucinol (DAPG), hydrogen cyanide, pyrrolnitrin or phenazines, also help protect bacteria against protist predation (Jousset et al., 2006). In addition, the presence of protists can induce the biosynthesis of the lipopeptidic surfactants massetolide and viscosin, which have been primarily investigated for their antimicrobial activities against plant pathogens (Andersen and Winding, 2004; Mazzola et al., 2009). Further, the exoprotease AprA inhibits various bacterivorous protists (Jousset et al., 2006) while also contributing to the suppression of plant-parasitic nematodes (Siddiqui et al., 2005). If there is a large degree of overlap between bacterial defense against protist predation and traits conferring plant protection, then introduction of soil protists could promote soil functionality and plant health. Increased selective pressure imposed by predators would thus coincide with increases in plant protective capabilities. A recent study by Asiloglu et al. (2020) further supports this idea: they showed that the application of soil protists enhanced the survival of the plant-beneficial bacterium *Azospirillum* sp. B510 in the rhizosphere of rice (*Oryza sativa* L.).

While highly attractive, the proposed link between predation resistance and plant beneficial activity still requires empirical verification to allow for the development of effective and predictable levels of soil function enhancement. In the present study, we therefore sought to (1) investigate the extent to which predatory pressures align with phylogenetic proximity for both predator and prey, and (2) test whether protist-bacteria interactions can be predicted as a function of bacterial traits known for their contribution to plant growth promotion, pathogen suppression and/or root colonization success. We scrutinized the interactions between seven soil *Pseudomonas* spp. described in relation to their plant-beneficial activity (Agaras et al., 2015) and six heterotrophic protists. The genus *Pseudomonas* was chosen as a model due to the well-known role of many of its members in plant growth promotion and protection (Haas and Défago, 2005; Lugtenberg and Kamilova, 2009), thus an extensive available literature and a high interest for application. Protist species were selected to cover three phylogenetic supergroups (Rhizaria, Excavata, and Amoebozoa) as well as the morphotype categories of amoeba, amoebo-flagellates and flagellates. The bacterial and protist isolates were cultivated in all pairwise predator-prey combinations, and the growth of both bacteria and protists were recorded and related to the characterized bacterial traits. We hypothesized that bacterial isolates harboring traits associated with pathogen suppression would be more resistant to protist predation.

## Materials and Methods

### Bacterial and Protist Isolates

We selected seven bacterial strains from a collection of *Pseudomonas* spp. isolated from Argentinian agricultural soils and previously characterized as described in Agaras et al. (2015). The selection comprises *Pseudomonas fluorescens* strain RBBP4, *Pseudomonas donghuensis* strain SVBP6, *Pseudomonas putida* strain SVMP4, *Pseudomonas asplenii* strain RPBP2 and three *Pseudomonas chlororaphis* strain SVBP8, strain SMMP3 and strain SVBP3 (see also Supplementary Table S1 for an overview and the density at the day of inoculation). Studying *Pseudomonas* spp. has the advantage to build upon an extensive literature that scrutinized its plant-beneficial activity, including both plant promotion and pathogen suppression, thus offering a vast array of available data regarding genetic and physiological traits (Walsh et al., 2001; Haas and Défago, 2005; Lugtenberg and Kamilova, 2009). The bacterial isolates were chosen to cover a range of plant-beneficial traits linked with plant growth promotion and disease suppression. In a previous greenhouse study, we showed that the plant-beneficial activity of these bacterial isolates was stimulated by the presence of the amoeba *Acanthamoeba castellanii* (Weidner et al., 2017) prompting further investigation on predator-prey interactions.

*Escherichia coli* OP50 was included in the setup to serve as a positive control for the growth of the protists. *E. coli* OP50 is routinely used as food source for our protist cultures. To our knowledge, *E. coli* OP50 does not possess any antagonistic activities against plant pathogens, nor any anti-predation strategies.

The protist isolates were selected to represent some of the main phyla of soil-dwelling free-living protists, while also including some closely related isolates (two Rhizaria, two Excavata, two Amoebozoa), and covering various morphotypes (two flagellates, two amoebo-flagellates, two amoeba; Table 1). Protists were isolated from a range of environments (clay soil, sandy soil and growth substrate) in the Netherlands and grown on *E. coli* OP50. The taxonomic assignment of the protists was obtained by extracting DNA from cultures of each protist isolate. Several pairs of general eukaryotic primers were used to facilitate the recovery of nearly full-length 18S rRNA gene sequences from each strain. Resulting sequences were subjected to BLASTn searches against NCBI GenBank (for more details see Gao, 2020, Chap. 3).

**TABLE 1:** Description of the protist isolates used in the present study.

**Code**	**Taxonomic assignment**	**Eukaryotic supergroup**	**Morpho-type**	**Origin**	**References**
C5D3	*Cercomonas lenta*	Rhizaria	Flagellate	Clay soil	DSM 32401^∗^
S24D2	*Cercomonas* sp.	Rhizaria	Flagellate	Sandy soil	Gao, 2020
P147	*Vannella* sp.	Amoebozoa	Amoeboid	Growth substrate	Gao, 2020
C13D2	*Acanthamoeba* sp.	Amoebozoa	Amoeboid	Clay soil	Gao, 2020
NL81	*Naegleria clarki*	Excavata	Amoebo-flagellate	Growth substrate	Gao, 2020
P145-4	*Naegleria clarki*	Excavata	Amoebo-flagellate	Growth substrate	Gao, 2020

Taxonomic assignment is as described in Gao (2020). The eukaryotic supergroups were assigned according to Adl et al. (2018). *The protist isolate Cercomonas lenta C5D3 *has been deposited in 2015 by ECOstyle BV at the Leibniz Institute DSMZ-German Collection of Microorganisms and Cell Cultures as Cercomonas lenta ECO-P-01 DSM 32401.*

### Growth Conditions and Preparation of Bacterial Isolates

All bacterial isolates were kept as frozen glycerol stocks (−80°C). Prior to the experiments, bacteria were grown on King’s B plates (KB; King et al., 1954), with one colony serving to initiate a new liquid culture in King’s B (28°C, 120 rpm, 14–15 h). For practical reasons, we worked with a modified KB recipe using potassium dihydrogen phosphate (KH_2_PO_4_); the pH of the solution was adjusted to 7.0. Bacterial cells were washed three times by centrifugation (9,500 g, 2 min) and resuspension in 0.9% NaCl. The pellets were eventually resuspended in Page’s Amoeba Saline, a diluted phosphate buffer used to grow protists (Page, 1976; hereafter referred to as PAS) and adjusted to an OD_600_ of 1.5. By plating a 10-fold serial dilution of the bacterial suspension, we estimated the cell densities for each isolate (Supplementary Table S1).

### Growth Conditions and Preparation of Protist Isolates

The protist cultures were routinely propagated supplemented with *Escherichia coli* OP50 as sole prey in PAS at 15°C, in the dark; fresh cultures were initiated once a month. *E. coli* OP50 was typically added at a density of *ca* 10^8^ cells mL^–1^. The protist stock cultures are thus usually a mixture of cyst and active individuals.

To obtain an active population for the co-cultures, we prepared protist culture as follows: stock protist cultures were washed three times by gentle centrifugation at 100 g for 10 min to remove spent medium, dead cells and potential contaminations. After centrifugation, the protists are concentrated in the lower part of the tube. Because they do not form any visible pellet, we only discard 75% of the volume before resuspending the cells in the same volume of PAS. Washed cultures were then amended with *E. coli* OP50 at a density of *ca* 10^8^ cells mL^–1^ to support protist growth. Protist cultures were incubated at 15°C in the dark for 3 or 5 days. The duration was adapted to each protist isolate with the aim to enable excystation and growth while avoiding new encystation.

To initiate the co-cultures, the obtained active populations of protists were washed as described in the previous paragraph, counted and adjusted to 10^3^ active individuals mL^–1^; note that despite our procedure the population of *Naegleria* sp. NL81 was already mostly encysted (Supplementary Table S2). To ensure that the protist inoculation was consistent across all wells, we estimated the protist density before, during, and after the inoculation procedure. The density was estimated by transferring a volume of 10 μL in Clear Polystyrene 96-Well Microplates with flat bottom (Corning 3370). The cells were enumerated over the full surface created by the drop on a monitor connected to an inverted microscope Nikon Eclipse TS 100 equipped with a DS Camera Control unit DS-L3 with DS-Fi2 camera head (relay lens: 0.7x) using the 20x objective (final magnification on the monitor: 275x).

Since the washing procedure does not allow for a complete elimination of *E. coli*, we plated a 10-fold dilution series of the washed protist solution on King’s B nutrient medium to estimate the number of cells transferred along with the protists (Supplementary Table S2); these remaining *E. coli* cells represented 1–10% of the total bacterial density in the co-cultures. To examine the potential influence of these residual *E. coli* cells on protist growth, we set up wells without any addition of prey cells. These wells are referred to as “No added cells.”

### Setup and Monitoring of the Cultures

Pure cultures and co-cultures (one bacterial isolate, one protist isolate) were prepared in Clear Polystyrene 96-Well Microplates with flat bottom (Corning 3370; see Supplementary Table S3 for the volume distribution for each well). Each combination was set up in five replicates. The location of each culture was randomized to take potential edge effects into account. Plates were sealed with Parafilm and incubated in the dark at 20°C for 5 days. The growth medium (2% King’s B, diluted in PAS) was chosen to mimic a low nutrient system. The OD_600_ was measured every day with a plate reader (SPECTROstar Nano, BMG Labtech) as indicator of bacterial density. Measured OD_600_ values were corrected for path length, so that depicted values correspond to a standard light path of 1 cm. Preliminary calibrations revealed that protist present in the wells did not significatively affect the optical density. Before each measurement, plates were briefly shaken (double orbital, 5 s at 500 rpm) to homogenize the cultures.

The co-cultures were also set up in triplicate in PAS to investigate the ability of the protist isolates to grow on the bacterial isolates under nutrient-limiting conditions. In case the protist would not grow on the bacteria in PAS, nor in 2% KB, it would indicate that the bacteria represent an inappropriate food source. On the other hand, if the protist does grow on the bacteria in PAS but not in 2%KB, this would indicate an active defense mechanism like the production of antibiotic compounds. An additional scenario might be observed, where the protist cannot grow on the bacterial isolate in PAS but can grow in 2% KB suggesting our system to be bottom-up regulated.

Protist density was estimated in a non-destructive manner after 1, 3, and 5 days of incubation. Encysted and active individuals were enumerated separately on three surface areas (264,000 μm^2^ per area), covering two non-central and one central location per well. The average of these three counts was then used to estimate the density per well. Cells were counted on a monitor connected to a Nikon Eclipse TS 100 inverted microscope with a phase contrast. We mainly used the 20x objective (final magnification on the monitor: 275x) but also the 40x objective (final magnification on the monitor: 550x) in cases where it was difficult to differentiate between active cells, cysts and/or cluster of bacterial cells. As all used organisms are living attached to the surface and not in suspension, protist concentration is expressed as individuals cm^–2^.

We chose to focus our data analysis on the third day after inoculation due to the specific growth pattern of the *Naegleria* spp. on *E. coli* OP50 in 2% KB and in PAS (Supplementary Figure S1). The population of both *Naegleria* spp. (P145-4 and NL81) showed an optimum density at day 3 before decreasing markedly at day 5, while the *Cercomonas* spp. (S24D2 and C5D3), the *Acanthamoeba* sp. (C13D2), and the *Vannella* sp. (P147) strains grew following similar patterns on the bacterial isolates at day 3 and 5 after inoculation. Noteworthy, even though *Naegleria* sp. NL81 cultures started with only cysts, excystation occurred rapidly, and the protists followed a similar growth pattern than the other *Naegleria* sp. used in this study (Supplementary Figure S1).

### Statistical Analyses

All data were analyzed using the version 3.4.3 of the open source statistical software R (R Core Team, 2017).

First, we investigated the growth of all protists combined on the different bacterial isolates in 2% KB. Note that we infer growth from density measurements, without incorporating maintenance and death as component of the density due to limitations of our methodology (amount of time points and ability to distinguish between living and dead cells); the same is true for the analysis of the bacteria. Due to zero inflation and overdispersion, we could not use a GLM assuming a Poisson distribution (Zuur et al., 2009). We decided to use the zero-inflated model hurdle or two-part for our data (Zuur et al., 2009). The hurdle model is comprised of two models: one model fits the abundance of the data, and the other model is a logistic regression reporting the probability of a non-zero count (presence/absence) (Zuur et al., 2009). We used the pscl:hurdle (Zeileis et al., 2008) function specifying the count model family to be negative binomial because of the observed overdispersion in our data. Using the base:summary function on the model, we extracted the significance of each explanatory variables (i.e., the bacterial isolates) to explain the observed protist densities.

We also investigated the growth of each protist separately on each bacterial isolate. The generalized and/or two-part models were not suitable for these analyses, potentially due to the lower number of data per group (five data points per group) and the high number of zeros for some groups. To correct for the heteroscedasticity of the data, we used a square root transformation. We ran an ANOVA analysis (stats:lm and base:summary) on the transformed data, using bacterial isolates as explanatory variable for the protist density at day 3 in 2% KB.

We computed a heatmap to show the protist density of each species in co-culture with each bacterial isolate (gplots:heatmap; Warnes et al., 2016). The protist density was centered and scaled per row, i.e., per protist isolate, to enable a visual comparison between species. The protist and the bacterial isolates are displayed according to their phylogenetic proximity.

Bacterial phylogenetic analyses were carried out with concatenated partial 16S rRNA, *rpoB* and *oprF* gene sequences (Mulet et al., 2010; Agaras et al., 2015). For this, we selected 510 nt within the 5′ region of the 16S rRNA gene (positions 110–619 in *Pseudomonas protegens* Pf-5, AJ417072) plus 480 nt within the 5′ region of the *rpoB* gene (positions 1,575–2,085 in *P. protegens* Pf-5; NC_004129.6) and 510 nt of the *oprF* gene (positions 263–742 in *P. protegens* Pf-5, NC004129). The corresponding concatenated sequences (1,490 bp) of the seven pseudomonads isolates were included in the analysis inferred by the Maximum Likelihood method based on the Kimura 2-parameter model (Kimura, 1980). Evolutionary analysis was conducted in the software MEGA7 (Kumar et al., 2016). All positions containing alignment gaps and missing data were eliminated (deletion option).

Phylogenetic analyses for protists were carried out using nearly full-length 18S rRNA gene sequences (see details in Gao, 2020). Maximum-Likelihood phylogenetic trees were constructed within SeaView Version 4 (Gouy et al., 2010). In order to assess the stability of the clades, phylogenetic analysis was performed based on Bayesian analysis using MrBayes 3.2 (Huelsenbeck and Ronquist, 2001). The evolutionary model was conducted under 6 General Time Reversible (GTR) substitution types with gamma-distributed rate variation across sites and a proportion of invariable sites.

We further investigated the effect of protists on the bacterial density using as proxy the OD_600_. We plotted the treatment mean of bacterial density against the treatment mean of protist density [log_10_(active cells cm^–2^ + 1); addition of a one because of the presence of zeros] at day 3 in 2% KB and computed a Spearman rank correlation (stats:cor.test). To further investigate this relationship, we ran an ANOVA analysis (stats:lm and base:summary) using protist isolates as explanatory variable for the bacterial density (OD_600_ values) at day 3 in 2% KB. We ran the analysis separately for each bacterial isolate. We computed a heatmap to show the bacterial density of each isolate exposed to each predator protist (gplots:heatmap; Warnes et al., 2016).

We then investigated correlations between the protist density and specific bacterial traits. Most data on bacterial traits were obtained from Agaras et al. (2015). The protocol and results of the drop collapse assay to identify biosurfactant production were reported for SVBP6 in Agaras et al. (2018); results for the other bacterial isolates can be found in the Supplementary Material of the present manuscript (Supplementary Table S4). We used Spearman rank correlations (stats:cor.test) to analyze the relation between protist density and the bacterial traits with counts or continuous data (i.e., number of inhibited fungi, inhibition of *Pythium*, HCN production in liquid medium, phospholipase relative activity in egg-yolk agar, exoprotease relative activity in milk agar, production of siderophore, solubilization of inorganic phosphorous, 1-aminocyclopropoane-1-carboxylate (ACC) deaminase activity, production of auxin indole-3-acetic acid (IAA), swimming, swarming and twitching motility). We performed point-biserial correlations (ltm:biserial.cor; Rizopoulos, 2006) to study the relation between the protist density and dichotomous data of the bacterial traits (i.e., presence/absence of the genes *phzF* for production of phenazines, *prnD* for pyrrolnitrin, and *pltB* for pyoluteorin, presence/absence of biosurfactant (drop collapse activity), and presence/absence of the N-acylhomoserine lactone (AHL) type of quorum sensing signals). All correlations were combined into one correlation matrix (corrplot:corrplot; Wei and Simko, 2017). We computed the statistical significance tests using stats:cor.test specifying the method to be Spearman or Pearson for the point-biserial correlation.

Using the Spearman rank correlation (stats:cor.test), we also investigated the correlation between the combined densities of all protist isolates at day 3, in 2%KB, and plant-beneficial related indexes proposed by Agaras et al. (2015), as well as the correlation between the combined bacterial densities at day 3 in 2%KB, and the indexes. The results were displayed using corrplot:corrplot. The indexes proposed by Agaras et al. (2015) are: the Biocontrol Potential Index (BPI, e.g., antibiotic genes, HCN production, lytic enzymes) and the Direct Growth Promotion Index (DGPI, e.g., P solubilization, IAA, ACC deaminase). The Colonization Potential Index (CPI) was constructed considering motility, quorum sensing, and biofilm activities separately from the rest. Each index was computed for every bacterial isolate based on a ratio between its activity value and the highest measurement in the set of tested isolates, normalized with the number of measured activities (see Agaras et al., 2015).

## Results

### Impact of Soil Pseudomonas on Protist Performance

**FIGURE 1:**
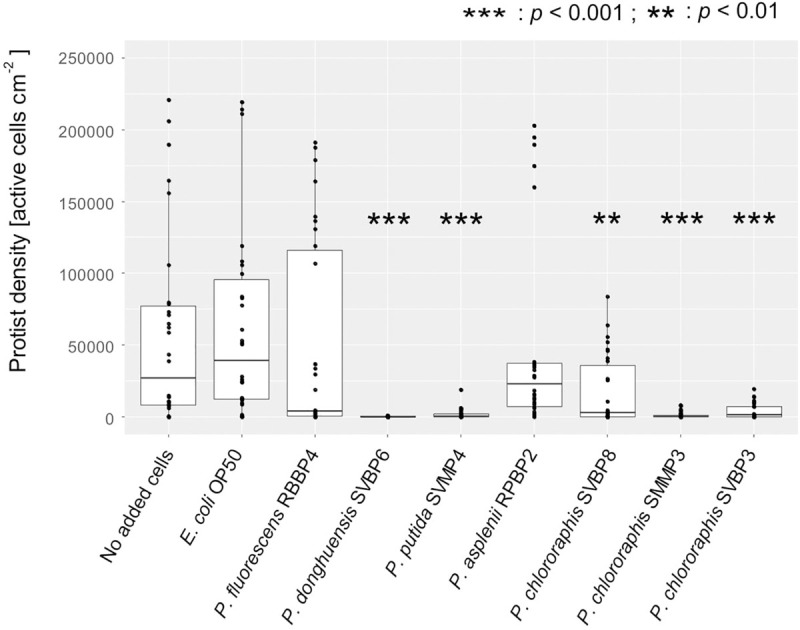
Active protist densities grown on different bacterial isolates (No added cells, *E. coli* OP50, and *Pseudomonas* spp.) at day 3, in 2% KB, shown for all protists together. Asterisks indicate significant differences compared to the control (protist grown on the *E. coli* OP50) reported from the binomial regression part of the hurdle model (see also Supplementary Table S5).

**FIGURE 2:**
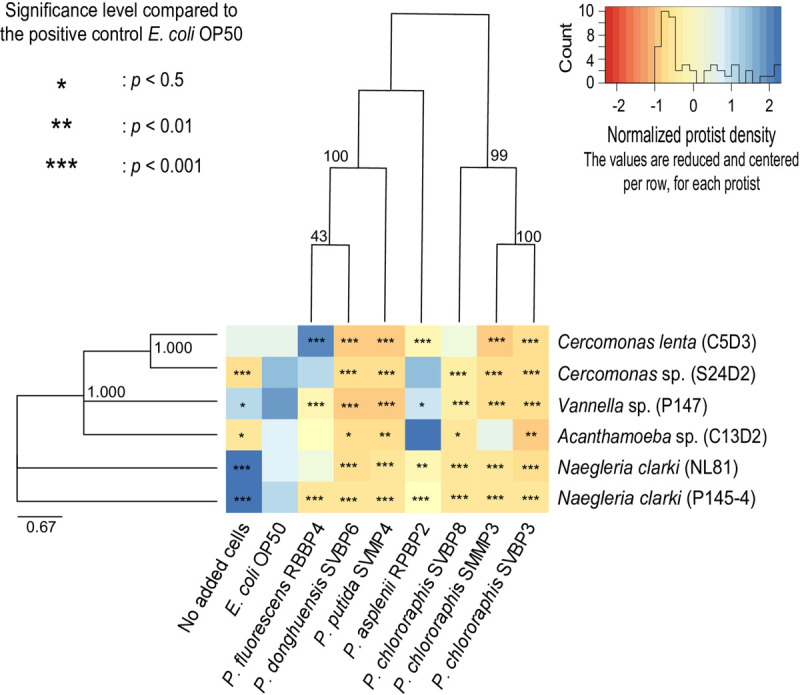
Active protist densities grown on each bacterial strain at day 3, in 2% KB, shown for individual predator-prey co-cultures. The different colors of the heatmap represent the normalized protist density on each bacterial isolate. White (corresponding to a value of 0) indicates the average density per protist isolate (per row). Orange indicates lower density compared to the average of a protist isolate and blue indicates higher density compared to the average of a protist isolate. Asterisks indicate level of significance in protist density grown on the given bacterial isolate relative to growth with *E. coli* OP50. The protist isolate and bacterial isolates are displayed based on their phylogenetic relatedness. Phylogenetic trees are based on the Maximum-Likelihood Method using the concatenated partial sequences from 16S rRNA, rpoB, and oprF genes for the pseudomonads and the 18S rRNA gene for the protist isolates.

Five of the seven pseudomonads significantly inhibited protist growth (Figure 1 and Supplementary Table S5). The isolates *P. donghuensis* SVBP6, *P. putida* SVMP4, and *P. chlororaphis* SVBP3 inhibited all six protist isolates, while the other bacterial isolates let at least one protist isolate grow to similar density compared to the positive control *E. coli* OP50 (Figure 2 and Supplementary Table S6 for the ANOVA table).

The bacterial ability to inhibit protist growth was only partially mirroring phylogenetic proximity. Indeed, all *P. chlororaphis* strains of the study inhibited the six protist isolates (Figure 2). However, the anti-predator potential of *P. donghuensis* SVBP6 is much more similar to that of *P. putida* SVMP4, compared to its closely related *P. fluorescens* RBBP4 (Figure 2).

Similarly, the growth patterns of the different protist isolates were only partially consistent with eukaryotic supergroups. For instance, the two *Cercomonas* spp. (Rhizaria) grew well on *P. fluorescens* RBBP4, while the Amoebozoa and Excavata showed an average growth or even lower growth (compared to *E. coli* OP50). Similarly, the two *Naegleria* spp. (Excavata), achieved their highest densities on the low density of *E. coli* OP50 given by the “no added cells” wells. In other cases, species-specificity was observed: for example, *Cercomonas* sp. S24D2 grew well on *P. asplenii* RPBP2, while *Cercomonas lenta* C5D3 did not. Similarly, *Naegleria clarki* NL81 grew well on *P. fluorescens* RBBP4, but *Naegleria clarki* P145-4 did not.

While we mainly focused on the co-cultures in 2% KB when analyzing predator-prey interactions, we briefly report here important and contrasting patterns observed in the nutrient limiting conditions of the PAS setup. In the co-cultures grown in PAS, *P. donghuensis* SVBP6 and two of the *P. chlororaphis* (SVBP3 and SMMP3) did not inhibit any of the protist isolates (see also Supplementary Table S7 and Supplementary Figure S2). In addition, *Naegleria* spp. formed cysts with all bacterial strains when in PAS, but not in the presence of the bacterial isolates *P. donghuensis* SVBP6, *P. putida* SVMP4 and the three *P. chlororaphis* SVBP3, SVBP8, and SMMP3 in 2% KB.

### Impact of Protist Isolates on Bacterial Performance

**FIGURE 3:**
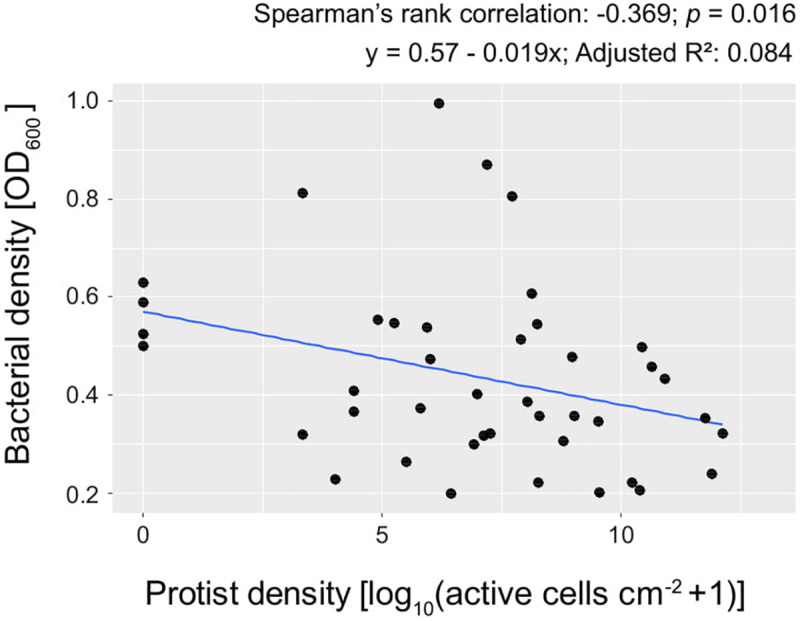
Bacterial density (OD_600_) plotted against protist density at day 3, in 2% KB. Each point represents the mean of 5 replicates.

The bacterial density was in general negatively correlated with protist density (Spearman’s rank correlation coefficient: −0.37, *p* = 0.016; Figure 3).

With reference to bacterial performance, we observed a clear distinction between those bacterial isolates that could inhibit the protist isolates as opposed to those that could not. Except for *P. chlororaphis* SMMP3, the bacterial isolates able to inhibit all protists were not affected by the presence of the predators. These bacterial isolates achieved similar optical density independent of the protist presence and identity (Table 2 and Supplementary Figure S3). The other bacterial isolates (*E. coli* OP50, *P. asplenii* RPBP2, and *P. fluorescens* RBBP4) were all influenced in their density by the presence of at least one protist isolate; we observed both lower and higher means compared to the control treatment (no protist) (Table 2 and Supplementary Figure S3).

**TABLE 2:** ANOVA table on bacterial density (expressed as OD_600_) after 72 h incubation in 2% KB, explained by using the protist presence presence as categorical variable [seven categories: no protist present, or one of the six protist isolated used in the study (Cercomonas spp., Acanthamoeba sp., Vannella sp., Naegleria spp.)].

	**ANOVA**		
**Bacteria**	***F*(6,28)**	***p-*value**	**adj. *R*^2^**
*E. coli* OP50	14.65	< 0.001	0.707***
*P. asplenii* RPBP2	21.52	< 0.001	0.784***
*P. fluorescens* RBBP4	8.57	< 0.001	0.572***
***P. chlororaphis* SVBP8**	1.15	0.362	0.025
***P. chlororaphis* SVBP3**	0.65	0.688	–0.065
***P. chlororaphis* SMMP3**	3.77	0.007	0.328**
***P. donghuensis* SVBP6**	1.26	0.308	0.043
***P. putida* SVMP4**	2.18	0.075	0.172

The bacterial isolates found to inhibit protist growth (Figure 1) are highlighted in bold. Statistical significance is highlighted for ***p < 0.001 and **p < 0.05.

### Correlation Between Plant-Beneficial Traits and Resistance to Predation

We further investigated the correlation between a suite of bacterial traits related to plant growth and health and protist density. The bacterial traits had been previously measured for each isolate by Agaras et al. (2015, 2018).

**FIGURE 4:**
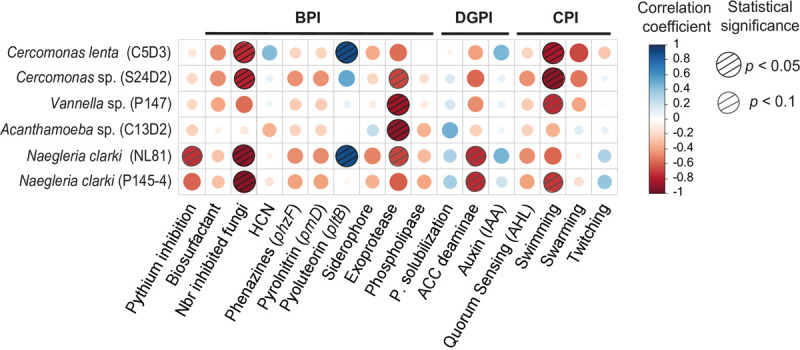
Correlation matrix between protist growth and selected prey bacterial traits. Red and blue dots indicate negative and positive correlations, respectively. Dashed circles are statistically significant. The different bacterial traits are grouped together according to the indexes: BPI, Biocontrol Potential Index; DGPI, Direct Plant Growth Promotion; CPI, Colonization Potential Index.

In general, bacterial traits associated with pathogen suppression showed negative trends with protist growth (Figure 4). The inhibition of fungal plant pathogens was for instance significantly negatively correlated with the growth of four protist isolates (*Cercomonas* spp. S24D2 and C5D3, and *Naegleria* spp. P145-4 and NL81; Figure 4 and Supplementary Table S8 for the statistical tests). The relative exoprotease activity shown in milk agar was further negatively correlated with the density of four assessed protist isolates (Figure 4 and Supplementary Table S8). Other traits (the inhibition of the oomycete *Pythium ultimum*, production of phospholipase, of biosurfactant, of hydrogen cyanide and of siderophores) showed a negative, yet only marginally significant trend with the growth of all protist isolates (Figure 4 and Supplementary Table S8). The genetic potential to produce antibiotics (phenazines, pyrolnitrin, pyoluteorin) was only negligibly correlated with protist growth inhibition; we even observed significantly positive correlations between the *pltB*-carrier (for production of pyoluteorin) and the density of *Cercomonas lenta* C5D3 and *Naegleria clarki* NL81 (Figure 4 and Supplementary Table S8).

We also detected positive trends between protist density and two direct plant growth promotion traits (inorganic phosphorus solubilization and auxin production). Surprisingly, ACC deaminase was negatively correlated with protist density (Figure 4 and Supplementary Table S8).

We further mainly observed negative correlations between protist density and bacterial traits related to root colonization. Especially swimming motility was associated with a low density of all protist isolates (Figure 4 and Supplementary Table S8).

**FIGURE 5:**
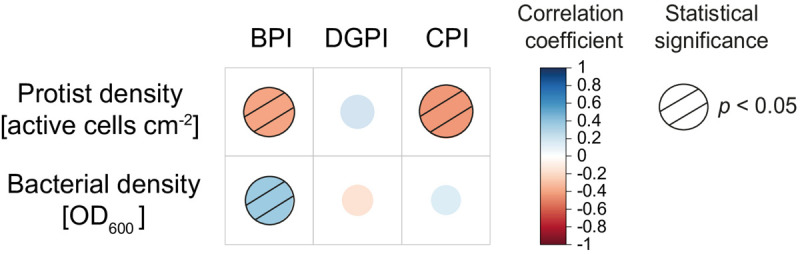
Spearman rank correlation between the combined protist density and the combined bacterial density with the different bacterial indices calculated by Agaras et al. (2015). Red and blue dots indicate negative and positive correlations, respectively. Dashed circles are statistically significant. Biocontrol Potential Index (BPI; e.g., antibiotic genes, HCN production, lytic enzymes), Direct Growth Promotion Index (DGPI; e.g., P solubilization, IAA, ACC deaminase), and Colonization Potential Index (CPI; motility, quorum sensing, biofilm).

Looking at the general patterns, the total protist density was significantly negatively correlated with the Biocontrol Potential Index (BPI) and the Colonization Potential Index (CPI). In contrast, the bacterial density (all isolates together) was positively correlated with the Biocontrol Potential Index (Figure 5).

## Discussion

We examined the extent of the overlap between resistance to predation and traits related to pathogen inhibition typically reported in rhizosphere bacteria. Because resistance to predation can be directly related to bacterial fitness, the observed overlap could explain the prevalence of plant-beneficial bacterial traits in the rhizosphere.

In our study, resistance to predation was found to be correlated with level of investment in plant-protective traits of *Pseudomonas* spp., a prevalent taxon in the rhizosphere. In contrast, predator-prey interactions generally could not be well predicted by the phylogenetic relatedness of either the microbial prey or predators. We therefore argue that protists may offer new approaches to support a pathogen-suppressive microbiome.

### Specificity of Predator-Prey Interactions

Bacterial isolates varied in their effect on the protist predators, from no inhibition to strong inhibition of all species tested. The bacterial isolates *Pseudomonas asplenii* RPBP2 and *Pseudomonas fluorescens* RBBP4 supported growth of most of the protist isolates. In contrast, the predator-resistant isolates *Pseudomonas donghuensis* SVBP6, *Pseudomonas putida* SVMP4, *Pseudomonas chlororaphis* SVBP3, SVBP8, and SMMP3 were highly effective at inhibiting the tested protist isolates. The predator-resistant bacterial isolates seem thus to harbor defense mechanisms effective against a range of organisms, spanning at least three eukaryotic supergroups. Such broad defense suggests non-specific mechanisms such as production of broad-range antibiotics or extracellular lytic enzymes (Whipps, 2001; Raaijmakers et al., 2002). Interestingly, protist inhibition only occurred in our 2% KB system, and not under nutrient-limiting conditions setup (i.e., PAS). This suggests that the bacterial isolates enable protist growth, but if enough nutrients are available, they reach higher densities and actively defend themselves provided they harbor the necessary genetical toolbox for anti-predator activity. This is further supported by the fact that all bacterial isolates reached higher densities in the 2% KB system compared to the PAS system [mean OD_600_ in 2%KB: 0.41, in PAS: 0.14; *t*_(__403__)_ = -19, *p* < 0.001; data not shown].

Although some closely related predator and prey strains acted similarly in our co-culture assays, phylogenetic proximity was not a strong predictor of protist feeding patterns. Preferential feeding of protists has previously been reported, but the contribution of taxonomy and/or phenotypic traits to this selectivity remain mostly unclear (Montagnes et al., 2008). For example, Pedersen et al. (2011) observed both a similar response for protists belonging to the same supergroup on secondary metabolite producing *Pseudomonas* spp. as well as a better resistance of the amoeboid taxa. However, Glücksman et al. (2010) showed that closely related and morphologically similar protists could have a very different impact on bacterial communities. Thus, even though some patterns can be generalizable to higher taxonomic level, very species-specific interactions occur as well and the prediction of the predatory effect of a protist species remains difficult. We are currently still lacking measurable traits of protists that might help to understand and potentially predict such interactions. In future research, consideration of protist traits such as cell flexibility, growth rate and prey density optimum in addition to the morphotype and taxonomy may help to define predictors of predator-prey interactions.

Surprisingly, no cysts were observed for any of the tested protist isolates when grown in the presence of the inhibitory bacteria. Encystation is a widespread survival mechanism used to help withstanding stressful conditions such as environmental extremes (Shmakova et al., 2016), and it has been shown to be induced by microbial compounds such as 2,4-diacetylphloroglucinol (DAPG), pyoluteorin (Jousset et al., 2006) and putrescine (Song et al., 2015). Bacterial compounds can, however, also adversely affect cyst formation and/or viability as reported for *Naegleria americana* with viscosin (Mazzola et al., 2009) and putrescine (Song et al., 2015). Adverse effects on encystation may therefore explain the absence of cysts in our study.

Bacterial density was negatively correlated with protist density, but, interestingly, the predation-resistant bacterial isolates were in general not influenced by exposure to protists. The isolates *P. donghuensis* SVBP6, *P. putida* SVMP4, *P. chlororaphis* SVBP3 and SVBP8 all achieved similar optical densities independent of the exposure to any predators. Our results are in line with previous work by Pedersen et al. (2009) where the predation-resistant *Pseudomonas protegens* CHA0 was not affected by the presence of either the flagellate *Cercomonas longicauda* or the nematode *Caenorhabditis elegans*. The bacterial isolates affected by the presence of predators showed both lower and higher densities compared to the no-protist control, in a species-specific manner (Supplementary Figure S3).

Based on the predator-prey interactions studied in the present study, we cannot predict species-specific interactions based only on phylogeny. While all *P. chlororaphis* spp. had a significant adverse effect to all protist isolates, *Acanthamoeba* sp. could still grow on *P. chlororaphis* SMMP3 and *Cercomonas* sp. C5D3 on *P. chlororaphis* SVBP8. In addition, the bacterial isolate *P. donghuensis* SVBP6 shared a much more similar inhibition pattern with *P. putida* SVMP4 than with its closer relative *P. fluorescens* RBBP4.

We speculate that the strong and broad anti-protist activity of the bacterial isolates *P. donghuensis* SVBP6, *P. putida* SVMP4, *P. chlororaphis* SVBP3, SVBP8, and SMMP3 is likely transferable to other predators. We expect that the ability to inhibit a broad range of predators would increase the survival and establishment of inoculated bacteria.

### Bacterial Traits Correlated to Predation Resistance

Bacterial traits related to pathogen suppression conferred a broad protection against protists. The ability of bacteria to resist predation can provide a competitive advantage compared to predation-susceptible bacteria. When exposed to predation and in mixture with other bacteria, *Pseudomonas protegens* CHA0 was shown to grow better compared to its isogenic *gacS* deficient mutant (Jousset et al., 2008) or compared to other *Pseudomonas* spp. described with lower predation resistance (Pedersen et al., 2009). Here we link such general resistance to previously reported measurable bacterial traits.

In particular, the ability of the bacterial isolates to inhibit pathogens (fungal pathogens and *Pythium ultimum*) strongly overlapped with their ability to inhibit the protist isolates. The combined inhibition of pathogens and predator protists has been reported in previous studies for various amoeba of the Amoebozoa supergroup (Andersen and Winding, 2004; Jousset et al., 2010; Novohradská et al., 2017), common soil flagellates from the Rhizaria and Excavata (Pedersen et al., 2010) as well as for the ciliate *Tetrahymena pyriformis* (Schlimme et al., 1999). Our study further supports this overlap by showing that members of the Amoebozoa, Rhizaria, and Excavata are all similarly inhibited by the same set of soil pseudomonads, and by identifying traits strongly correlated with the observed inhibition.

Interestingly, the production of lytic enzymes such as proteases or lipases was a better predictor of general anti-protist activity than antimicrobial compounds. Lytic enzyme production is one of the factors driving the biocontrol activity of fungi (Segers et al., 1994; Bonants et al., 1995) and bacteria (Dunne et al., 1997; Siddiqui et al., 2005) against pathogen and pests. In a study comparing functional mutants of *Pseudomonas protegens*, Jousset et al. (2006) also reported a contribution of the extracellular protease AprA to the toxicity against protists. Phospholipase are another group of lytic enzymes known to promote cytolysis of macrophages (Schmiel and Miller, 1999). Because macrophages and amoeba share many similarities (Escoll et al., 2013; Novohradská et al., 2017), phospholipase could contribute to protist inhibition. The present work suggests that exoenzymes contribute to a general protection mechanism against protist predation.

The potential for antibiotic production was, in contrast, only marginally correlated with protist inhibition. For instance, bacteria able to produce biosurfactants, which have previously been proposed to supress protists (Mazzola et al., 2009; Song et al., 2015), only had a weak, non-significant effect on *Cercomonas* spp. Hydrogen cyanide production (HCN) was also only weakly related to the resistance to predators. This is in line with the low toxicity reported for *Acanthamoeba castellanii*, which could survive exposure of up to 5 mM KCN (Jousset et al., 2010). Regarding antibiotic genes, the presence of the *prnD* gene (pyrrolnitrin) and the *phzF* gene (for phenazines) were only weakly associated with protist inhibition. This result coincides with the previously reported small contribution of pyrrolnitrin to protist predation resistance (Müller et al., 2013). The potential contribution of phenazines, known to be toxic for nematodes (Cezairliyan et al., 2013), remains unknown. More surprising is the positive correlation between the *pltB* genes and *Cercomonas lenta* C5D3 and *Naegleria* sp. NL81, which was in contrast to previous studies reporting adverse effects of pyoluteorin against protists (Winding et al., 2004; Jousset et al., 2006). An additional candidate antibiotic compound, 7-hydroxytropolone, was recently reported for *P. donghuensis* SVBP6 to be at the origin of the broad-spectrum *in vitro* antifungal activity displayed by this bacterium (Muzio et al., 2020). Tropolone and products containing tropolonoid motifs display antimicrobial activities attributed to their metal-chelating and redox properties (Guo et al., 2019) and derivatives have shown some anti-protozoan activities (Ren et al., 2003).

Production of ACC deaminase was associated with the inhibition of the two *Naegleria* spp. of our study. The production of ACC deaminase by bacteria typically reduces plant ethylene content, thereby promoting plant growth in the absence of stress (Glick, 2014; Ravanbakhsh et al., 2018). A direct adverse effect of ACC deaminase against protists is rather unlikely. The mechanism behind the observed correlation is unclear and could be due to a covariate not included in our study. Nonetheless, if ACC deaminase is consistently correlated with negative protist density, bacteria producing this enzyme could be selected in a community exposed to protist predation.

Traits linked to root colonization and bacterial fitness, such as quorum sensing molecules and motility, were negatively associated with protist density. The production of N-acylhomoserine lactones (AHLs) was only weakly related to protist inhibition, despite previous reports of quorum-sensing related traits for antagonistic interactions (Jones et al., 1993; Peng et al., 2018). In contrast, swimming motility was strongly correlated with the inhibition of both *Cercomonas* spp., *Naegleria clarki* P145-4 and *Vannella* sp. High swimming speed has indeed been reported to provide bacteria with efficient protection against predation (Matz and Jürgens, 2005).

Highly motile bacteria with low biocontrol activity could thus, nevertheless, have a selective advantage under predator pressure. Indeed, the bacterial isolate *P. putida* SVMP4 with low biocontrol activity but a high motility could efficiently escape predation of all protist isolates. In addition to exoprotease and biosurfactant production reported for *P. putida* SVMP4, motility could also contribute significantly to the resistance to predation of this strain. Showing the exact opposite trend, *P. chlororaphis* SVBP8 reported with an overall strong biocontrol activity, but only medium production of exoprotease and medium swimming motility, was not as successful as *P. putida* SVMP4 in inhibiting the protist isolates. The contribution of motility to resist predation is particularly relevant because biocontrol and plant-growth promotion activity presents a potential trade-off (Agaras et al., 2015). It is, however, worth noting that while motility has been shown to increase survival in aquatic system (Matz and Jürgens, 2005) where possibilities for motility are high, similarly to our experimental setup, the benefits of motility may differ in a more heterogenous medium such as the soil depending on additional variables such as structure and moisture (Erktan et al., 2020).

Our data support the hypothesis that bacterial isolates with functional traits related to pathogen suppression can also better resist predation. Further, we highlight the importance of swimming motility to escape predation. Recent results from field assays have also demonstrated that, within this set of probiotic *Pseudomonas*, those with high BPI values showed the highest effect on maize and wheat productivities, over three consecutive seasons and in different locations (Agaras et al., 2020). The correlation of BPI values and the resistance to predation supports the idea that biocontrol traits confer high adaptability and survival in complex environments, such as soil and rhizosphere, allowing the isolates to better display their plant growth promotion.

## Conclusion and Perspectives

In the present study, we show that several bacterial traits associated with plant growth and health are correlated with bacterial resistance to protist predation. This relationship appears to be rather general, offering better predictive capabilities than the phylogeny for either the prey or the predator. We show an important overlap between resistance to predation and pathogen suppression. Our correlation analysis especially suggests an important contribution of extracellular lytic enzymes such as exoproteases and highlights the important contribution of motility traits to resist predation.

Extrapolation to the complex soil system from our liquid system should be approached with caution, but we suggest that application of specific protist species can promote targeted functions in the soil microbiome. Depending on the resident bacterial community, the application of the *Cercomonas* spp. and *Naegleria* spp. has thus the potential to support bacteria with high biocontrol activity against fungi, while the application of the two Amoebozoa of our study might support exoprotease production. Not only biocontrol activity could be promoted, but also traits linked to direct plant growth promotion as illustrated by the association between ACC deaminase and inhibition of *Naegleria* spp. as well as the important contribution of motility to resist predation.

In conclusion, we suggest that understanding the linkage between bacterial fitness (here predation resistance) and traits related to pathogen inhibition allows strategically promoting beneficial microbiome functions.

## Data Availability Statement

The raw data supporting the conclusions of this article will be made available by the authors, without undue reservation, to any qualified researcher.

## Author Contributions

The idea of the present research follows previous work done in collaboration between BA, EL, CV, AJ, and SW. SW and AJ supervised the study. NA performed the experiment, did the analysis and wrote the first draft of the manuscript. ZG identified the protist isolates and performed the phyologenetic tree analysis. BA isolated and identified the bacterial isolates, measured the various traits, and performed the phylogenetic tree analysis. GK was mainly involved in the discussion of the results and the revision of the manuscript. All co-authors have provided feedbacks and comments that substantially improved the manuscript.

## Conflict of Interest

The authors declare that the research was conducted in the absence of any commercial or financial relationships that could be construed as a potential conflict of interest.
