# Supplementary material for: Biocontrol Traits Correlate With Resistance to Predation by Protists in Soil Pseudomonads
Source: Front Microbiol. 2020 Dec 15;11:614194. doi: 10.3389/fmicb.2020.614194 (PMC7769776; doi:10.3389/fmicb.2020.614194)
Supplement: Supplementary file 1 [file Data_Sheet_1.pdf]

Supplementary Material

1 Supplementary Material

1.1 Supplementary tables

Table S1: Description of the bacterial isolates used in this study. The taxonomic assignment is as described in Agaras et al. (2015). The estimated densities at the day of inoculation (d0) of the present study are given in the table.

**Strain code**	**Taxonomic assignment**	**Origin**	**Reference**	**Density at d0 [CFUs mL^{-1}]**
OP50	*Escherichia coli*	-	Brenner (1974); NCBI:txid637912	7.00×10^{8}
RBBP4	*Pseudomonas fluorescens*	Soybean rhizosphere	Agaras *et al.* (2015)	2.33×10^{8}
SVBP6	*Pseudomonas donghuensis*	Bulk soil	Agaras *et al.* (2015 & 2018)	4.00×10^{8}
SVMP4	*Pseudomonas putida*	Bulk soil	Agaras *et al.* (2015)	1.83×10^{8}
RPBP2	*Pseudomonas asplenii*	Maize rhizosphere	Agaras *et al.* (2015)	5.00×10^{8}
SVBP8	*Pseudomonas chlororaphis*	Bulk soil	Agaras *et al.* (2015)	2.17×10^{8}
SMMP3	*Pseudomonas chlororaphis*	Bulk soil	Agaras *et al.* (2015)	2.67×10^{8}
SVBP3	*Pseudomonas chlororaphis*	Bulk soil	Agaras *et al.* (2015)	3.83×10^{8}

Table S2: Estimation of the protist density and remaining Escherichia coli OP50 cells in the cultures used for predator-prey experiments.

**Protist isolate**	**Active protists [cells mL^{-1}]**	**Encysted protists [cells mL^{-1}]**	**OP50 density [CFUs mL^{-1}]**
Cercomonas lenta C5D3	7500	0	9.33×10^{6}
Cercomonas sp. S24D2	7500	0	3.33×10^{6}
Vannella sp. P147	5000	0	1.32×10^{7}
Acanthamoeba sp. C13D2	1000	0	8.33×10^{6}
Naegleria clarki NL81	0	7500	7.67×10^{6}
Naegleria clarki P145-4	2500	10	9.83×10^{6}

Table S3: Composition of the different wells for the co-inoculation.

	2%KB [µl]	Bacterial solution [µl] [1]	Protist solution[µl] [2]	Final volume [µl]
**Co-cultures**	125	15	10	150
**Bacterial cultures**	135	15	0	150
**Protist cultures** ***(No bacteria added)***	140	0	10	150
**Blank**	150	0	0	150

[1] Bacterial densities were at 1 to 7 x 10^8 CFUs per mL.

[2] Protist densities was adjusted to $10^{2\text{-}3}$ active individuals per µL. The protist NL81 was inoculated as cysts.

Table S4. Biosurfactant activity of culture supernatants of Pseudomonas isolates used in the present study. The taxonomic assignment for the Pseudomonas *is as described in Agaras et al. (2015). The protocol of the drop collapse assay to identify biosurfactant production is reported in Agaras et al. (2018).*

		**Growth medium**	
**Strain code**	**Bacterial isolate**	**OS-glucose**	**King's B**
RBBP4	*P. fluorescens*	-	-
SVBP6	*P. donghuensis*	-	-
SVMP4	*P. putida*	+	+
RPBP2	*P. asplenii*	-	-
SVBP8	*P. chlororaphis*	-	-
SMMP3	*P. chlororaphis*	+	+
SVBP3	*P. chlororaphis*	-	-

*Table S5: Estimated coefficients and significance tests for the negative binomial part and the logistic part of the hurdle model (pscl::hurdle and stats::summary) used to compare protist growth on each bacterial isolates at day 3, in 2%KB. We use as reference level for the model the protist density grown on remaining E. coli OP50 (No for No added bacterial cells). S.E.=standard error. Statistical significance is highlighted for p<0.001 with "***", for p<0.01 with "**", for p<0.05 with "*" and for p<0.1".".*

**Abundance - Negative binomial regression part**					
	**Coefficient**	**S.E.**	**z value**	***p* value**	**Statistical significance**
No (Intercept)[1]	11.024	0.244	45.168	< 0.001	***
OP50	0.048	0.339	0.142	0.887	
RBBP4	0.038	0.359	0.106	0.916	
RPBP2	-0.264	0.342	-0.773	0.440	
SMMP3	-3.451	0.384	-8.996	< 0.001	***
SVBP3	-2.439	0.363	-6.714	< 0.001	***
SVBP6	-5.157	0.445	-11.599	< 0.001	***
SVBP8	-1.094	0.355	-3.079	0.002	**
SVMP4	-3.139	0.363	-8.642	< 0.001	***
**Presence/absence - Logistic regression part**					
	**Coefficient**	**S.E.**	**z value**	***p* value**	
No (Intercept)[2]	2.639	0.732	3.606	< 0.001	***
OP50	15.927	1963.405	0.008	0.994	
RBBP4	-1.253	0.863	-1.452	0.146	
RPBP2	0.728	1.253	0.581	0.561	
SMMP3	-2.093	0.824	-2.539	0.011	*
SVBP3	-1.450	0.850	-1.706	0.088	.
SVBP6	-3.045	0.821	-3.707	< 0.001	***
SVBP8	-1.030	0.881	-1.169	0.242	
SVMP4	-1.450	0.850	-1.706	0.088	
Number of iterations in BFGS optimization: 19					
Log-likelihood: -2365 on 19 Df					

[1] *The coefficients in the first row of the negative binomial regression part states if the model of our reference level (here protists grown on remaining* E. coli *OP50, no added bacterial cells) is significantly different from 0.*

[2] *The coefficient in the first row of the logistic regression part gives the probability of a non-zero count of our reference level.*

Table S6: ANOVA table on protist density (square root transformed) after 72h incubation in 2% KB, expressed as a function of bacterial isolates identity.

	ANOVA		
**Protist**	**F(8,45)**	***p* value**	**adj. R^2**
C5D3	145.4	< 0.001	0.96
S24D2	17.33	< 0.001	0.75
P147	32.01	<0.001	0.85
C13D2	4.45	0.001	0.39
NL81	27.72	< 0.001	0.83
P145-4	220	< 0.001	0.98

*Table S7: Estimated coefficients and significance tests for the negative binomial part and the logistic part of the hurdle model (pscl::hurdle and stats::summary) used to compare protist growth on each bacterial isolates at day 3, in PAS. We use as reference level for the model the protist density grown on E. coli OP50. S.E.=Standard Error. Statistical significance is highlighted for p<0.001 with "***", for p<0.05 with "*" and for p<0.1". ".*

**Count model coefficients (truncated negative binomial with log link)**					
	**Coefficient**	**S.E.**	**z value**	***p* value**	**Statistical significance**
OP50 (Intercept)	10.889	0.296	36.765	< 0.001	***
No added bacteria	-3.185	0.457	-6.967	< 0.001	***
RBBP4	-0.005	0.425	-0.012	0.990	
SVBP6	-0.637	0.425	-1.499	0.134	
SVMP4	-1.079	0.419	-2.575	0.010	*
RPBP2	-0.790	0.425	-1.859	0.063	.
SVBP8	-0.925	0.425	-2.178	0.029	*
SMMP3	-0.629	0.448	-1.404	0.160	
SVBP3	-0.245	0.439	-0.557	0.577	
Log(theta)	-0.457	0.102	-4.460	< 0.001	***
**zero hurdle model coefficients (binomial with logit link)**					
	**Coefficient**	**S.E.**	**z value**	***p* value**	
OP50 (Intercept)[2]	19.570	4179.000	0.005	0.996	
No added bacteria	-18.610	4179.000	-0.004	0.996	
RBBP4	-16.730	4179.000	-0.004	0.997	
SVBP6	-17.430	4179.000	-0.004	0.997	
SVMP4	0.000	5910.000	0.000	1.000	
RPBP2	-16.730	4179.000	-0.004	0.997	
SVBP8	0.000	5996.000	0.000	1.000	
SMMP3	-18.310	4179.000	-0.004	0.997	
SVBP3	-17.960	4179.000	-0.004	0.997	
Signif. codes: 0 '***' 0.001 '**' 0.01 '*' 0.05 '.' 0.1 ' ' 1					
Number of iterations in BFGS optimization: 16					
Log-likelihood: -1653 on 19 Df					

[1] *The coefficients in the first row of the negative binomial regression part states if the model of our reference level (here* E. coli *OP50) is significantly different from 0.*

[2] *The coefficient in the first row of the logistic regression part gives the probability of a non-zero count of our reference level.*

Table S8: Correlation coefficients and statistical test for the correlations between protist density (day 3, in 2%KB) and selected prey bacterial traits (Fig. 4). Only the treatments with p-values under 0.1 (yellow) or under 0.05 (red) are shown. The abbreviation "Nbre inhib. fungi" stands for "number of inhibited fungi".

**protist**	**Correlation type**	**bacterial trait**	**df**	***P* value**	**Corr Coefficient**
C5D3	Spearman	Nbre inhib. fungi	5	0.0477	-0.7594
C5D3	Pearson (point-biserial)	*pltB*-carrier	5	0.0067	0.8937
C5D3	Spearman	Swimming	5	0.0212	-0.8289
S24D2	Spearman	Nbre inhib. fungi	5	0.0424	-0.7709
S24D2	Spearman	Exoprotease	5	0.0938	-0.6786
S24D2	Spearman	Swimming	5	0.0068	-0.8929
P147	Spearman	Exoprotease	5	0.0137	-0.8571
P147	Spearman	Swimming	5	0.0522	-0.75
C13D2	Spearman	Exoprotease	5	0.0068	-0.8929
NL81	Spearman	Nbre inhib. fungi	5	0.0058	-0.8994
NL81	Spearman	Pythium inhib.	5	0.0626	-0.7298
NL81	Spearman	Exoprotease	5	0.0938	-0.6786
NL81	Pearson (point-biserial)	*pltB*-carrier	5	0.0073	0.8897
NL81	Spearman	ACC deaminase	5	0.0522	-0.75
P145-4	Spearman	Nbre inhib. fungi	5	0.0036	-0.9178
P145-4	Spearman	ACC deaminase	5	0.0522	-0.75
P145-4	Spearman	Swimming	5	0.0713	-0.7143

1.2 Supplementary figures

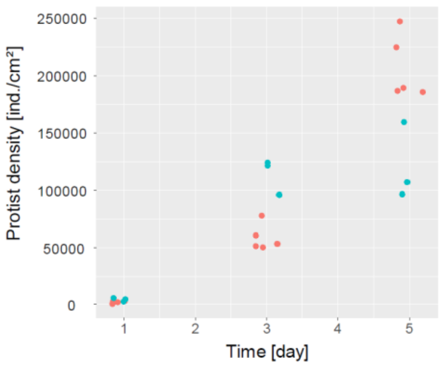

(A) *Cercomonas lenta* C5D3 grown on *E. coli* OP50

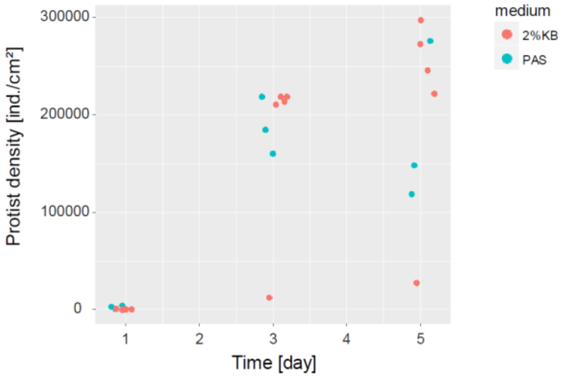

(B) *Cercomonas* sp. S24D2 grown on *E. coli* OP50

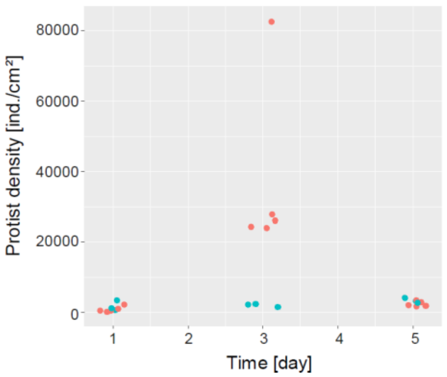

(C) *Naegleria clarki* NL81 grown on *E. coli* OP50

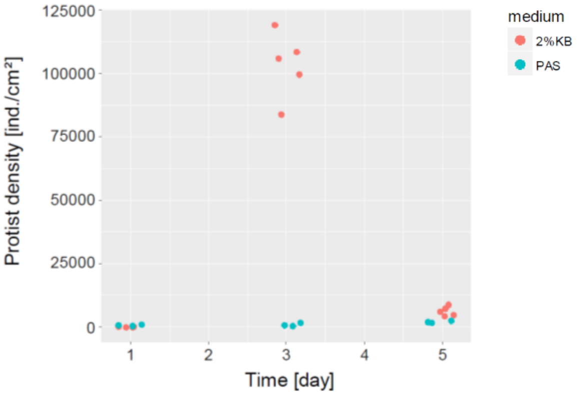

(D) *Naegleria clarki* P145-4 grown on *E. coli* OP50

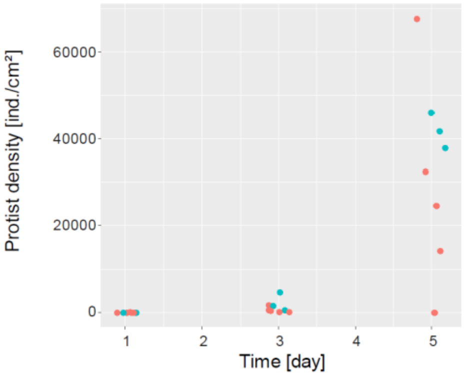

(E) *Acanthamoeba* sp.C13D2 grown on *E. coli* OP50

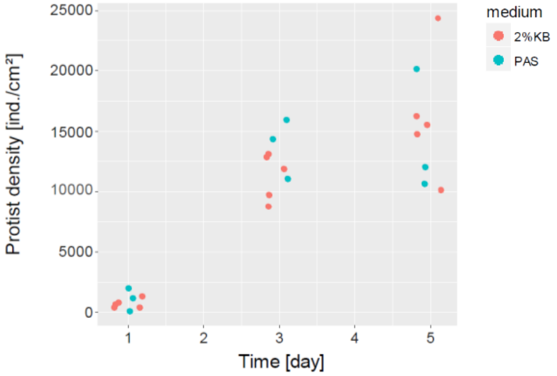

(F) *Vannella* sp. P147 grown on *E. coli* OP50

Figure S1 Temporal protist growth on E. coli OP50 in 2%KB and PAS – one graph per protist. Day 1 corresponds at day 1 after inoculation.

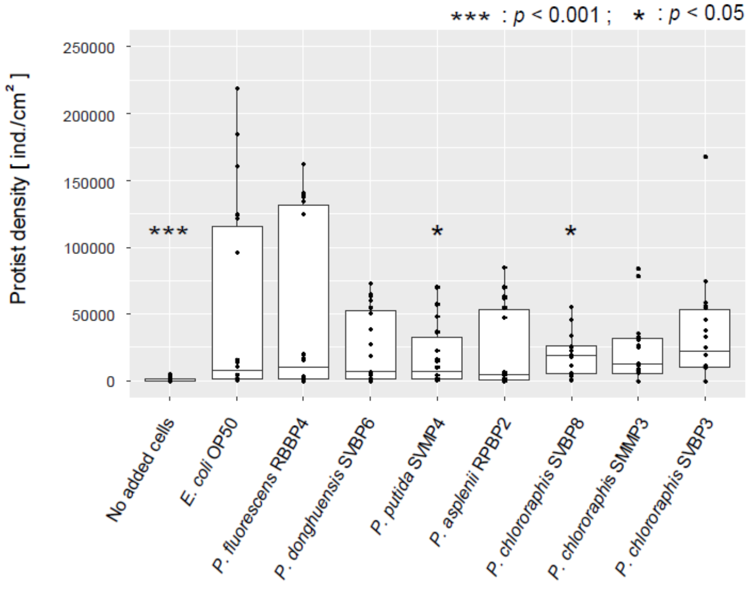

(A) General effect of the bacteria on the protists

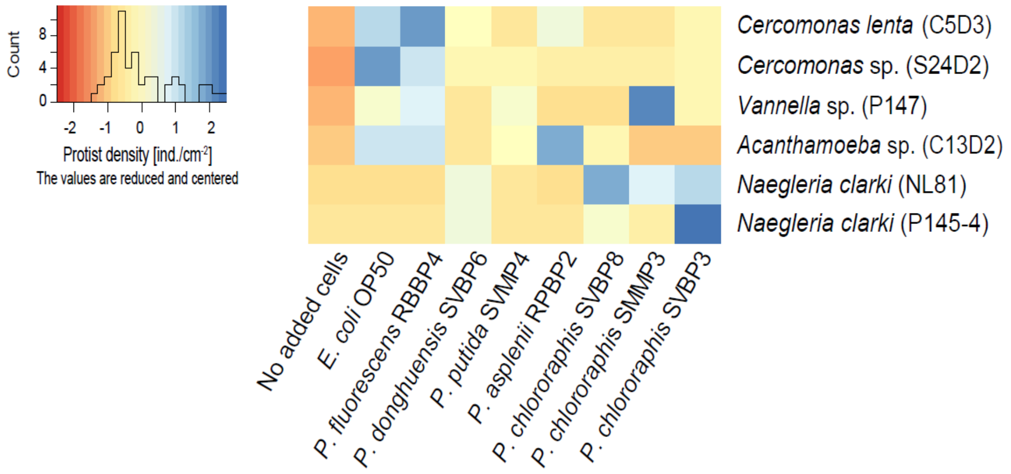

(B) Species-specific effect of each bacteria on each protist

Figure S2: Active protist densities grown on different bacterial isolates (E. coli, No added cells and OP50, and Pseudomonas spp.) in PAS, shown for all protist isolates together (A), and individual predator-prey co-cultures (B). Asterisks indicate significant differences compared to the control (protist grown on E. coli OP50) reported from the negative binomial regression part of the hurdle model. The different colors of the heatmap represent the normalized protist density on each bacterial isolate; orange indicating lower density (i.e. lower growth compared to the row average) and blue indicating higher density (i.e. higher growth compared to the row average) than the overall mean (for each protist).

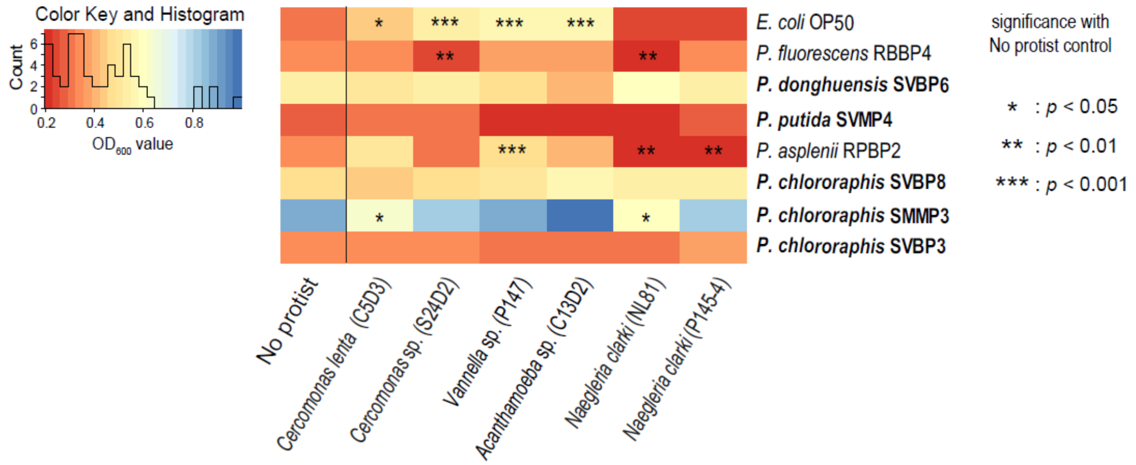

Figure S3: Mean optical densities measure at 600nm (OD_{600}) for each bacteria in 2% KB for individual predator-prey co-cultures. Significant differences compared to the control group (no protist) are highlighted with asterisk, based on the ANOVA analysis (base::summary). The five bacteria shown to inhibit all protists are highlighted in bold.

References

Agaras, B. C., Iriarte, A., and Valverde, C. F. (2018). Genomic insights into the broad antifungal activity, plant-probiotic properties, and their regulation, in *Pseudomonas donghuensis* strain SVBP6. PLoS ONE 13, e0194088. doi:10.1371/journal.pone.0194088.

Agaras, B. C., Scandiani, M., Luque, A., Fernández, L., Farina, F., Carmona, M., et al. (2015). Quantification of the potential biocontrol and direct plant growth promotion abilities based on multiple biological traits distinguish different groups of *Pseudomonas* spp. isolates. Biol. Control 90, 173–186. doi: 10.1016/j.biocontrol.2015.07.003

Brenner, S. (1974). The genetics of *Caenorhabditis elegans*. Genetics 77, 71–94.
